# Long-Term Growth in Phenylketonuria: A Systematic Review and Meta-Analysis

**DOI:** 10.3390/nu11092070

**Published:** 2019-09-03

**Authors:** Fatma Ilgaz, Alex Pinto, Hülya Gökmen-Özel, Julio César Rocha, Esther van Dam, Kirsten Ahring, Amaya Bélanger-Quintana, Katharina Dokoupil, Erdem Karabulut, Anita MacDonald

**Affiliations:** 1 Faculty of Health Sciences, Department of Nutrition and Dietetics, Hacettepe University, 06080 Ankara, Turkey; 2 Department of Dietetics, Birmingham Children’s Hospital, Birmingham B4 6NH, UK; 3 Center for Health Technology and Services Research (CINTESIS), 4200-450 Porto, Portugal; 4 Centro de Genética Médica Dr Jacinto de Magalhães, Centro Hospitalar Universitário do Porto, 4099-028 Porto, Portugal; 5 Centro de Referência na área das Doenças Hereditárias do Metabolismo, Centro Hospitalar Universitário do Porto—CHP EPE, 4099-001 Porto, Portugal; 6 Beatrix Children’s Hospital, University of Groningen, University Medical Center, 9700 RB Groningen, The Netherlands; 7 Department of PKU, Kennedy Centre, 2600 Glostrup, Denmark; 8 Enfermedades Metabolicas Servicio de Pediatria Hospital Ramon y Cajal, 28034 Madrid, Spain; 9 Department of Metabolism and Nutrition, Dr. von Hauner Children’s Hospital, University of Munich, 80337 Munich, Germany; 10 Faculty of Medicine, Department of Biostatistics, Hacettepe University, 06080 Ankara, Turkey

**Keywords:** phenylketonuria, hyperphenylalaninemia, growth, anthropometrics, weight, height, z-scores

## Abstract

There is an ongoing debate regarding the impact of phenylketonuria (PKU) and its treatment on growth. To date, evidence from studies is inconsistent, and data on the whole developmental period is limited. The primary aim of this systematic review was to investigate the effects of a phenylalanine (Phe)-restricted diet on long-term growth in patients with PKU. Four electronic databases were searched for articles published until September 2018. A total of 887 results were found, but only 13 articles met eligibility criteria. Only three studies had an adequate methodology for meta-analysis. Although the results indicate normal growth at birth and during infancy, children with PKU were significantly shorter and had lower weight for age than reference populations during the first four years of life. Impaired linear growth was observed until the end of adolescence in PKU. In contrast, growth impairment was not reported in patients with mild hyperphenylalaninemia, not requiring dietary restriction. Current evidence indicates that even with advances in dietary treatments, “optimal” growth outcomes are not attained in PKU. The majority of studies include children born before 1990s, so further research is needed to show the effects of recent dietary practices on growth in PKU.

## 1. Introduction

Phenylketonuria (PKU; OMIM 261600) is a rare inherited metabolic disease characterized by the absence of the liver enzyme phenylalanine (Phe) hydroxylase (PAH; EC 1.14.16.1) that converts Phe into tyrosine. The absence of this enzyme leads to elevation of blood Phe levels. If left untreated, irreversible neurological damage may occur due to the accumulation of Phe and its metabolites in the brain [1,2].

Based on pre-treatment blood Phe levels, patients are classified into three different phenotypes that require treatment: mild PKU with pre-treatment Phe levels of 360–600 μmol/L, moderate PKU with pre-treatment Phe >600–1200 μmol/L, and classical PKU with pre-treatment Phe >1200 μmol/L [2]. Patients presenting with a pre-treatment blood Phe level <360 µmol/L do not require treatment and are described as mild hyperphenylalaninemia (mHPA) [2,3]. A low Phe diet with natural protein restriction and supplementation with a synthetic Phe-free/low-Phe protein substitute is the standard treatment in PKU, although more recent alternative or adjunct therapies such as the use of tetrahydrobiopterin (BH4), or large neutral amino acids (LNAA’s) are prescribed for certain subgroups of patients. Dietary treatment is commenced following detection by newborn screening, preferably in the first two weeks of life in order to maintain blood Phe levels within a safe target range and to achieve optimal neurological development [3]. The severity of dietary restriction varies according to the residual activity of PAH enzyme (or phenotype), which influences individual Phe tolerance. Most patients with classical PKU tolerate less than 10 g natural protein (<20 mg/kg/day Phe), and most permitted natural protein sources are derived from plant sources such as fruits and vegetables [3,4,5]. For nutritional adequacy, the remaining protein requirements are usually provided by a Phe-free/low-Phe protein substitute supplemented with vitamins, minerals, and essential fatty acids [4]. Special low-protein foods (SLPF) provide energy and aid adherence by adding variety [5,6]. Milder phenotypes are likely to tolerate more natural protein (20–50 mg/kg/day Phe) and may respond to BH4 treatment, which allows some relaxation of natural protein intake.

There has been a considerable progress in dietary practices of PKU since its first introduction by Bickel and colleagues in 1951 [7]. In the early years of treatment, protein requirements were not fully understood, and the use of unpalatable low-Phe protein hydrolysates with inadequate nutritional composition resulted in poor adherence and unfavorable outcomes [8]. As the main objective of the treatment was the protection of brain from harmful effects of increased blood Phe levels, early treatment protocols used very restrictive diets, particularly during infancy and early childhood [9,10]. In the 1960s and 1970s, many PKU centers stopped dietary treatment as early as age 4 to 8 years in children, when it was believed that the brain development was essentially complete [11]. The age of continuing either a strict or relaxed diet was gradually extended, and in the early 1990s, lifetime dietary treatment was recommended in the UK, which was later reinforced by the European PKU Guidelines [3,12].

Lifelong treatment prevents neurocognitive impairment and abnormal executive functioning and helps maintaining mental health [3,13]. Over time, recommendations on target Phe levels and dietary protein intakes have changed [3]. There have also been efforts to improve the palatability and increase the availability of protein substitutes and SLPFs. The first Phe-free L-amino acid supplements were introduced in the 1970s, and since then, more acceptable protein substitutes with different presentations (e.g., powder, tablets, shakes), supplemented with micronutrients and essential fatty acids, have been developed [14,15,16,17]. However, both the nutritional composition and availability of these products significantly vary between countries [17].

Despite the improvements in dietary treatment of PKU, lifelong adherence is challenging, and there are concerns regarding the long-term use of a semi-synthetic low-Phe diet [18]. Early reports have suggested that initial treatment protocols caused growth impairment in children with PKU compared to healthy controls [19,20,21,22,23,24,25]. Although normal growth has been documented in more recent studies, which were mostly conducted in patients who were born after 1990s and who had good metabolic control [26,27,28,29], there are still some reports of reduced final height or suboptimal growth following adolescence, which was influenced by gender or disease phenotype [30,31,32].

Overall, there is ongoing debate about the impact of disease and treatment on long-term growth in PKU. The aims of this systematic review were: (1) to investigate if a Phe-restricted diet affects long-term growth in patients with PKU compared to normal populations, (2) to compare growth of patients with PKU treated by a Phe-restricted diet with mHPA patients who did not require dietary treatment, and (3) to determine if there are any growth gender differences between males and females.

## 2. Materials and Methods

This study was conducted by using the Preferred Reporting Items for Systematic Reviews and Meta-Analyses (PRISMA) current guidelines [33]. The protocol was developed by authors and registered to PROSPERO with the record number CRD42018110779.

### 2.1. Literature Search

A systematic literature review was performed in 4 electronic databases including PubMed, Web of Science, Scopus, and Central Cochrane Library. The following keywords were used in the PubMed search query: (“Phenylketonurias” [MeSH Terms] OR “Phenylketonurias” [All Fields] OR (“Phenylketonuria” [All Fields] AND “PKU” [All Fields]) OR “Phenylketonuria PKU” [All Fields]) AND (“Phenylketonurias” [MeSH Terms] OR “Phenylketonurias” [All Fields] OR “Hyperphenylalaninemia” [All Fields]) AND (“Growth and development” [Subheading] OR (“Growth” [All Fields] AND “Development” [All Fields]) OR “Growth and development” [All Fields] OR “Growth” [All Fields] OR “Growth” [MeSH Terms]). For the remaining three databases, these main terms were customized. We limited our search to English, Spanish, Italian, Portuguese, and French languages. The last search was completed on the 21 September 2018.

### 2.2. Study Selection

The PICO (population, intervention, comparison, outcomes) method was applied to formulate the review question, and to determine the eligibility criteria. All retrospective and prospective longitudinal studies, randomized-controlled trials, and case-control studies conducted in patients with PKU being treated with a Phe-restricted diet from all age groups, and with a minimum of two years of follow up were included. Preclinical studies (in vitro and in vivo studies conducted on cell cultures or animals), cross-sectional studies, reviews, case reports, abstracts and thesis, and studies without a clear definition of the dietary treatment or with insufficient growth data were excluded. Studies were also considered for exclusion if the study population included the following: (1) patients with maternal PKU, (2) patients with a late diagnosis of PKU, (3) untreated PKU patients whose dietary treatment was not started within the first two months of age, (4) patients with a diagnosis of tetrahydrobiopterin (BH4) deficiency, and (5) patients treated by sapropterin or Pegvaliase.

Two independent reviewers (F.I. and A.P.) screened titles and abstracts according to eligibility criteria. All potentially relevant articles were identified for full-text review. Disagreements were resolved by consensus or through discussion with a third author (A.M.).

### 2.3. Outcome Measures

The primary outcomes were anthropometric measurements or indexes related to physical growth including body weight, height/recumbent length, and body mass index (BMI). Secondary outcome measures were birth weight, head circumference, and measures of metabolic control (e.g., blood Phe levels).

### 2.4. Data Extraction

Data was collected by two independent authors (F.I. and A.P.) using a standardized data extraction form. Information obtained from all included studies was (1) study characteristics (authors, publication year, country, duration, and design of the study), (2) description of population (sample size, gender, age, and ethnic origin), (3) description of dietary treatment (time of diet initiation, level of Phe-restriction, types of Phe-free/low-Phe protein substitutes, dietary natural protein and Phe intakes, total protein intake, and duration of follow-up), and (4) outcomes (weight, height or length, BMI, birth weight, head circumference, body composition, blood Phe control, and parental growth). Growth data, expressed as both age-specific z-scores and/or as mean (±SD) value, was extracted from tables. If the growth data was only available in figures, open source software Plot Digitizer (version 2.6.8, General Public License, Ankit Rohatgi, Austin, TX, USA) was used. We corresponded with five authors of papers to obtain further information.

### 2.5. Quality Appraisal

Two authors (F.I. and E.K.) independently assessed the quality of evidence of the included studies by using the Grading of Recommendations Assessment, Development and Evaluation (GRADE) approach [34]. The GRADE ranks for risk of bias, inconsistency, indirectness and imprecision were “not serious,” “serious,” and “very serious,” and for publication bias as “not likely,” “likely,” and “very likely.”

### 2.6. Risk of Bias Assessment

Two independent reviewers (F.I. and E.K.) assessed the selected articles for risk of bias by using “The Risk of Bias in Non-Randomised Studies of Interventions (ROBINS-I)” assessment tool [35]. This tool was developed by the Cochrane Bias Methods Group and assesses internal validity. There are seven specific bias domains in the tool, including (1) confounding, (2) selection of participants, (3) classification of interventions, (4) deviations from intended interventions, (5) missing data, (6) measurement of outcomes, and (7) selection of reported results. Signaling questions were provided to help assessors decide the overall assessment for each domain. Risk of bias was rated as 0—no information; 1—low risk; 2—moderate risk; 3—serious risk; and 4—critical risk.

### 2.7. Data Analysis

The main objective of this systematic review was to assess how growth differs between PKU children compared with non-PKU control groups (e.g., healthy children, healthy siblings, or mHPA patients who have a normal diet). From 13 included articles, all studies measured weight and height or length (in children <2 years of age), except one study by Hoeksma et al. [36] that did not evaluate weight. Five studies also measured BMI, and head circumference was reported in seven studies. Body composition was investigated in only one study.

There was heterogeneity between studies in terms of presentation of growth data, but the most frequently used method was the z-score system [19,20,22,26,27,28,31,32,36,37]. Growth data was presented only with mean anthropometric values in three studies [8,38,39], and only two studies provided both [20,31]. As most of the included studies used z-scores, which is widely accepted for presentation and interpretation of anthropometric data with several advantages (e.g., evaluation of growth by combining gender and age groups) [40], the studies only showing mean values were excluded from this meta-analysis. We also excluded six articles due to missing or insufficient data (e.g., standard deviations, sample size too small). Overall, the meta-analysis was performed with the remaining three articles [20,31,32], and the excluded studies were evaluated qualitatively. For the interpretation of growth results, a mean z-score value of “zero” or “close to zero” was considered as “similar growth” between patients with PKU or mHPA and healthy population. The values between the two cut-off points of “−2SDs” and “+2SDs” were interpreted as “normal” range for growth [40].

The secondary objective was to determine the differences between growth of male and female patients with PKU. Two studies [20,31] reported genders separately. However, growth data was only limited to height-for-age z-scores (HAZ) in the first study [20] since body weight was expressed as “weight-for-height z-scores.” Therefore, gender difference could not be evaluated due to insufficient data.

BMI-for-age z-scores were only available in one study [31], as well as head circumference data [20]. We, therefore, only compared weight-for-age (WAZ) and HAZ of patients with PKU and control groups. The duration and the frequency of follow-up were also different between studies. In the study by Schaefer et al. [20], patients with PKU were followed from birth to six years of age with six-month intervals. The remaining two studies [31,32] evaluated patients with PKU from birth until 18 years of age, and the frequency of measurements ranged between six months to one year. In Thiele et al. study [32], dietary treatment was interrupted in 27 patients at a median age of seven years (range: 6.0–15.0). Since this could be a confounding factor, WAZ data between the ages of six and 18 were not included in the meta-analysis. Growth data of mHPA patients were only available in two studies [31,32]. Clinical visits for this group were scheduled less frequently after 12 years in one study [31], so WAZ and HAZ were evaluated in the meta-analysis between 1 to 12 years of age only. A pooled analysis could not be conducted on BMI, head circumference, and body composition due to lack of data.

Heterogeneity between studies was calculated by I^2^ statistic. I^2^ value of 25%, 50% and 75% were considered as low, medium and high heterogeneity, respectively. Given the high heterogeneity level between studies, a random-effects model was used to calculate pooled estimates with the “metafor” package of R software (version 3.5.1, R foundation for statistical computer, Vienna, Austria) [41]. A *p* value less than 0.05 was considered statistically significant.

## 3. Results

### 3.1. Study Selection

**Figure 1:**
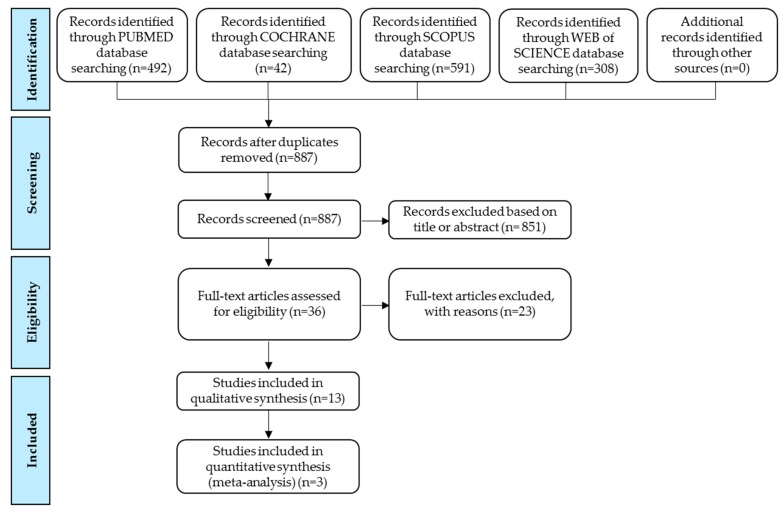
Study selection process according to Preferred Reporting Items for Systematic Reviews and Meta-Analysis (PRISMA) flow chart.

Figure 1 summarizes the study selection process. A total of 1433 articles were identified from four electronic databases. After the initial screening, 851 articles were excluded based on title or abstracts, and 36 citations were selected for full-text review. Finally, 13 eligible articles were included in the systematic review, and three articles were included in the meta-analysis.

### 3.2. Study Characteristics

Study characteristics of the included studies are presented in Table 1. Ten studies were conducted in Europe [19,20,22,26,27,31,32,36,37,39], one in Australia [28], and two in the United States [8,38]. Six of the studies collected longitudinal prospective data [8,19,20,28,37,39], and seven were retrospective [22,26,27,31,32,36,38]. Seven studies were published from 1983 to 1995 [8,19,20,22,37,38,39], and the remaining six were published from 2005 to 2017 [26,27,28,31,32,36]. A similar distribution was observed according to when the data was collected, such that in seven out of thirteen studies data collection was carried out from the early 1960s to the end of the 1990s [8,19,20,22,36,38,39]. Only four studies had more recent data collected from 2008 to 2014 [26,27,28,32]. Some authors did not specify the data collection period [31,37]. In three studies, only classical patients with PKU were included [8,37,38]. The phenotype of PKU was not specified in five studies [19,20,22,28,36], and the remaining studies included mixed phenotypes [8,26,27,31,32]. The sample sizes varied from 16 to 505 patients. Duration of follow-up ranged between two to 18 years. Most studies used national reference comparison growth data from healthy children. One study compared the data of patients with PKU with healthy siblings [23] (Table 2). Additional data regarding timing of diet initiation, description of interventions, metabolic control of patients and parental growth were presented in supplementary tables (Supplementary Materials Tables S1 and S3).

### 3.3. Systematic Review of Key Findings

An overview of the key findings of included studies is given in Table 2.

#### 3.3.1. Growth at Birth in PKU and mHPA

Weight and length-for-age z-scores at birth were evaluated in eight studies. Birth weight was not different from healthy controls in the majority of studies [8,20,26,27,31,32]. However, two studies showed that children with PKU were smaller at birth [19,22]. Birth length results were inconsistent as half of the studies showed that length at birth was normal or close to reference population [22,26,27,31], while others found that children with PKU were shorter [19,20,32,36]. In seven studies that evaluated head circumference [19,20,22,36,37,38,39], it was normal [22,38,39] or lower [19,20,36] than reference values at birth, but it gradually increased and reached expected values after the first year of life [19,36,37]. There was a gender difference in head circumference growth, as one study showed that it decreased by 0.4 SD in boys during the first year of life, compared to girls who showed minor change [20]. During follow-up head circumference in boys remained lower than reference population by 0.3 SD, but it remained at zero in girls. 

Only four studies evaluated growth at birth in patients with mHPA [26,27,31,32]. Birth weight was similar to reference population in all studies. Only one study found that mHPA children were shorter than healthy children at birth, but still within normal range (−0.49 SD) [26]. No study reported the head circumference at birth in mHPA.

#### 3.3.2. Long-Term Growth in PKU

There was a notable linear growth impairment after birth in children with PKU compared to reference groups. In eight of 13 studies, length/height was shorter, particularly during the first three years of life [8,19,20,22,31,32,36,37], in puberty [32], and on reaching adulthood [31]. There was height catch-up in only three studies [20,22,37]. In one study, a relaxed low-Phe diet at eight years led to improvement in height [22]. In boys, there was also evidence that height growth was more compromised and catch-up growth was delayed [20,32]. Height was found to be similar to reference population in four studies [26,27,28,37]. One study showed a deviation from target height in female and male PKU patients by −3 and −5 cm, respectively [32], but others did not find any differences [26,27]. A study by Kindt et al. [39] primarily investigated the long-term effects of two different protein recommendations on growth. The authors concluded that outcomes were satisfactory in both groups, but the sample size was low (*n* = 16).

Longitudinal weight measurements from seven studies showed that it was similar to reference [19,20,22,26,27,28,38] or higher during adolescence [26]. Four studies showed a lower weight for age [8,31,32,37], particularly during the first two years of life [31,37], and in classical PKU [8]. Five studies investigated BMI and none of them found a significant difference for children with PKU compared to reference [26,27,28,31,32]. Overweight rates were also below the reference values in both PKU and mHPA patients [31]. However, one study associated severe phenotype with overweight at the end of puberty [26].

#### 3.3.3. Long-Term Growth in mHPA

The few studies that evaluated growth in mHPA consistently showed that weight [26,27,31,32], height/length [26,27,31] and BMI [26,27,31,32] were close to reference population, except for Thiele et al. [32], who found that height z-scores were significantly lower in mHPA patients compared to healthy children during the first six years.

#### 3.3.4. Associations between Growth, Dietary Intakes and Blood Phenylalanine Control

A few studies investigated the association of growth with blood phenylalanine control [8,20,31,37] and dietary intakes (e.g., Phe, total protein, natural protein, calorie intakes) [8,28,31,36]. Four studies investigating the relationship between blood Phe levels and growth parameters did not show any significant correlation [8,20,31,37]. However, both weight and height were positively associated with blood Phe levels in a recent study with a large sample size (*n* = 505) and 18 years follow-up [31].

Only three studies investigated the effects of protein intake (total, natural and protein substitute) on growth [28,31,36]. Hoeksma et al. [36] found that head circumference was positively correlated with natural and total protein intakes, but not with protein equivalent intake from protein substitute. Additionally, there was no association between energy or protein intakes with height. Two other studies by Aldamiz-Echevarria et al. [31] and Evans et al. [28] failed to show a significant association between total protein, natural protein, or protein substitute intakes with anthropometric measurements. However, the latter found a negative correlation between fat mass (%) and total protein, natural protein, and protein substitute intakes. Evans et al. [28] also concluded that a safe protein:energy ratio of 3.0–4.5 g protein/100 kcal may support optimal growth. However, patients on BH4 treatment were evaluated together with diet-only treated patients in the correlation analysis, adding another variable to data interpretation.

### 3.4. Quality Appraisal

The results of quality assessment are presented in Table 3. Inconsistency of results was the only reason for downgrading.

### 3.5. Assessment of Risk of Bias

Risk of bias was evaluated by using ROBINS-I tool (Table 4). Overall, 12 of 13 studies were rated as “moderate risk of bias”, and only one study [22] was rated as “critical risk of bias” due to bias arising from deviations from intervention.

### 3.6. Meta-Analysis

**Figure 2:**
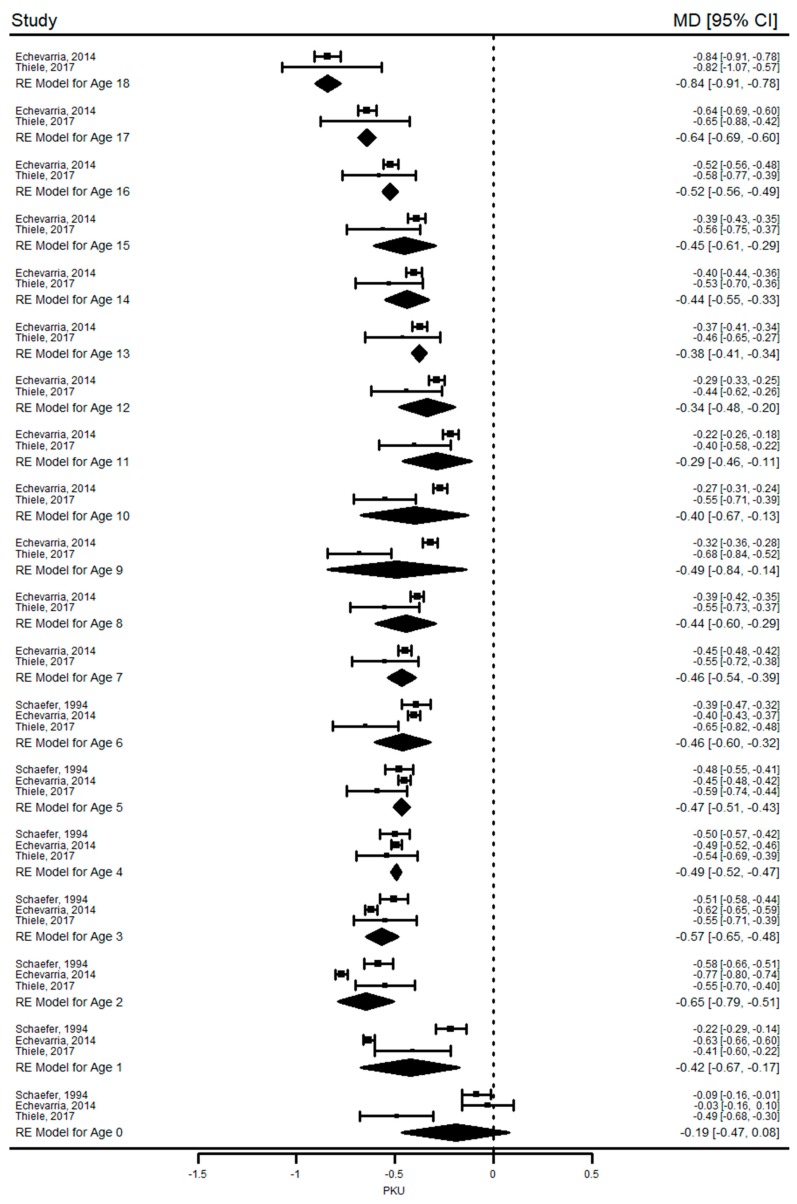
Comparison of mean difference height-for-age z-scores of patients with PKU compared to healthy population from birth to 18 years of age. Abbreviations: PKU: Phenylketonuria; RE: Random effects; MD: Mean difference; CI: Confidence interval.

**Figure 3:**
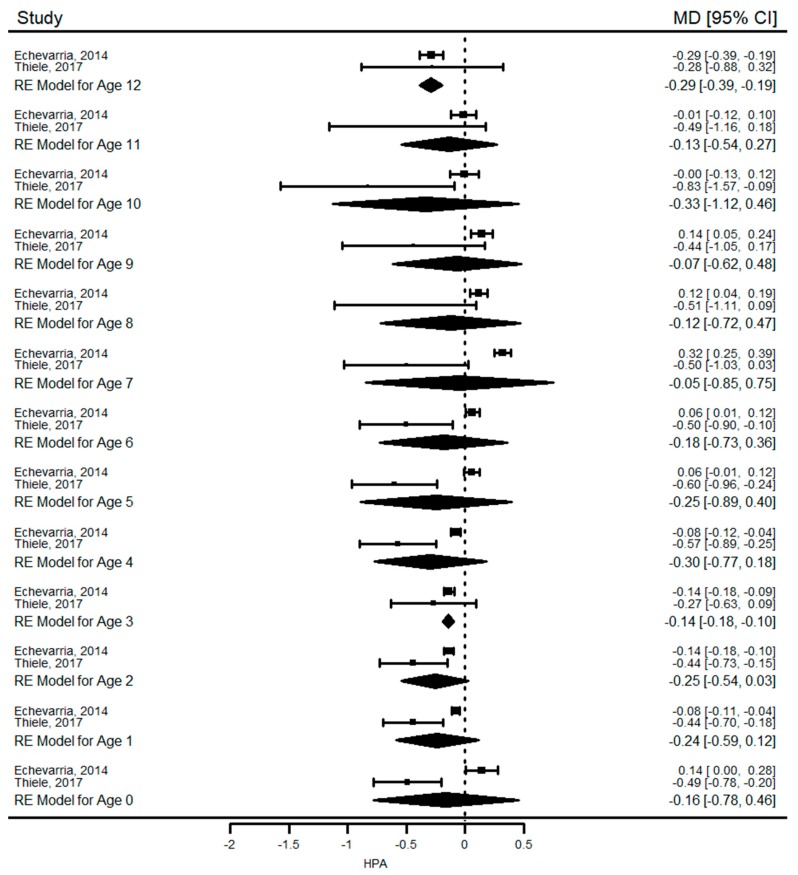
Comparison of mean difference height-for-age z-scores of patients with mHPA compared to healthy population from birth to 12 years of age. Abbreviations: HPA: mild-hyperphenylalaninemia; RE: Random effect; MD: Mean difference; CI: Confidence interval.

A pooled analysis of HAZ was performed with three studies (Table 5 and Figure 2) and WAZ with two studies (Table 6). HAZ of children with PKU was similar to those of healthy children at birth and at six months. After six months, children with PKU were significantly shorter than healthy controls until the end of adolescence. WAZ of PKU patients was not significantly different than healthy controls at birth, one year, and five years of age, but it was significantly lower in patients with PKU between 2–4 years of age. HAZ and WAZ were similar between mHPA and healthy children in most of the time points (Table 7 and Table 8 and Figure 3).

According to *I*^2^ values, heterogeneity between studies for height was generally ‘high’, and ‘low to moderate’ for weight. Time of recruitment (study period), country of study, sample size and duration of follow-up was different between studies. Gender distribution was similar but there was no information about phenotype distribution in two studies.

## 4. Discussion

This is the first systematic review and meta-analysis investigating the impact of a Phe-restricted diet on long term growth in patients with PKU. The primary aim was to determine if children with PKU could achieve a normal growth similar to healthy children from birth until adulthood. The effects of gender, disease severity (phenotype), metabolic control, and nutritional intake on growth were also evaluated.

Our results show that growth (height and weight) in PKU was similar to healthy children at birth and during infancy but they were significantly shorter and had lower weight for age than reference population during the first four years of life. We could not perform a long-term analysis for weight, but reduced height growth was observed in PKU until the end of 18 years of age when the mean difference in HAZ between PKU patients and controls reached its highest value of −0.8 SD. In children with mHPA, a pooled analysis of two studies revealed that growth was not significantly affected. Another finding of this systematic review was that mean HAZ and WAZ of children with PKU and mHPA were within the normal ranges in almost all included studies (mean difference between the z-scores was consistently close to or less than 1 SD). Overall, these results suggest that children with PKU have not attained their growth potential compared with healthy peers.

This meta-analysis and previous studies [8,19,20,21,22,23,31,32,36,37] consistently showed an impairment of growth in children with PKU during the first three years of life, when growth is mainly determined by nutritional factors. Later, genetics or hormonal status (e.g., growth hormone, thyroid hormones, insulin-like growth factor I, insulin-like growth factor binding protein, sex hormones) impact growth during later childhood and adolescence [42,43]. Several hypotheses have been suggested for the possible causes of suboptimal growth outcomes in PKU but studies addressing the effects of hormonal status, bone age, or genetics (parental growth) on growth in PKU failed to show any associations [20,32,44]. Therefore, faltering growth in young children with PKU has been mostly attributed to the use of a semi-synthetic low-Phe diet, commenced within the first few weeks of life. This is further supported by studies demonstrating no impact on growth in children with mHPA.

Despite substantial data on growth in PKU, the effects of dietary factors or metabolic control on growth outcomes have been little studied [8,20,23,28,30,31,32,36,44,45,46]. No relationship was found between blood Phe levels and anthropometric measurements [8,20,23,31,37,44,46]. Higher Phe intake was associated with better growth in one study [31], but this was not confirmed by others [8,44]. Inadequate energy intake was eliminated as a cause of impaired growth, as a Phe restricted diet does not limit energy from carbohydrates and fats [44]. There was also inconsistency between studies examining the effects of total protein, natural protein and protein equivalent intake from protein substitutes on growth outcomes. Considering earlier studies demonstrated unsatisfactory growth when total protein intake met the recommended dietary allowance (RDA) for general population [19,20], a higher protein intake (largely from Phe-free substitutes) was recommended to help achieving optimal growth [47]. This increment in total protein intake was associated with normal growth in most [26,27,30,45,48], but not all studies [31,32,44].

In our systematic review, we observed that mean total protein intake for a given age was higher in studies that showed optimal growth in children with PKU [26,27,28,36] compared to studies with suboptimal growth results (Supplementary Materials Table S2) [20,31,32]. One possible reason may be that protein prescriptions were different, as shown in a recent study comparing total protein prescriptions from different countries in Europe [49]. The authors concluded that there was variation according to region, with the highest median amount prescribed in Northern and Southern Europe, followed by Eastern and finally Western Europe [49]. Our results were similar, such that the mean total protein intakes were lower in studies conducted in Western Europe that showed suboptimal growth [20,32] compared to studies in Southern Europe with optimal outcomes [26,27]. Interestingly, the results of two recent studies from different centers in Southern Europe (Spain) presented inconsistent results. Growth was similar to healthy Spanish population in one study [26], but lower in the other [31]. Since the mean total protein intake in the first study showing adequate growth was 2–3 times higher than the latter study with impaired growth results (Supplementary Materials Table S2), one could speculate that different protein prescriptions may have led to the contrasting growth outcomes even in the same country or population.

Protein quality, rather than enhancement of total protein intake alone, may result in better growth outcomes or body composition. The effect of the type of protein on growth has been explored. Most studies failed to demonstrate an association between anthropometric measurements, natural protein [28,31,44] or protein substitute intake [28,31,36]. However, there has been evidence of a positive correlation between natural protein intake and head circumference (but not height) within the first three years of life [36]. Moreover, higher natural protein intake was positively associated with fat-free mass, and negatively correlated with fat-mass in two other studies [28,45]. We did not include studies conducted on patients treated with BH4, which is also associated with increased natural protein intake according to patient’s tolerance. However, evidence from literature about the effects of BH4 treatment on growth has shown inconsistent results [50,51,52]. Some of these studies included very small sample sizes and short follow-up periods. Therefore, it is too early to draw conclusions about the long-term effects of BH4 treatment and concomitant increase in natural protein intake on growth in PKU.

The impact of low biological efficiency of protein substitutes has also been explored. Low-Phe/Phe-free protein substitutes constitute the majority of dietary protein intake in children with PKU. It is well-established that ingestion of L-amino acids leads to rapid absorption, increased oxidation and poor nitrogen retention compared to intact proteins [53], especially when taken in large single doses rather than small frequent doses [54,55]. Low biological efficiency of L-amino acids in protein substitutes, poor adherence to the timing and dosage recommendations may compromise growth in young children who have increased protein turnover due to faster growth rates and who are more susceptible to any protein depletion than adults [56]. However, this has not been investigated in the studies included in this systematic review, so it is not possible to associate the metabolic efficiency of protein substitutes with growth outcomes.

Poor adherence is also an important issue which may possibly affect growth in PKU [57]. It has been well-established that adherence to the low-Phe diet deteriorates with age, especially during adolescence and adulthood [10,57,58,59,60]. Interestingly, this is the period in our study (11 to 18 years) in which patients were significantly shorter compared with reference values after catching up on differences from early childhood. It was not possible to establish an association between dietary adherence and growth from the results of this systematic review and meta-analysis due to lack of patient adherence rates (the mean percentage of patients with blood Phe levels within the target ranges). Additionally, the results were conflicting; there was still evidence of impaired growth despite good metabolic control, whereas better growth was achieved with poor adherence due to relaxation of the diet.

Being overweight is very common in the general population worldwide. In this systematic review, BMI in both children with PKU and mHPA was similar to healthy controls during the follow-up, and overweight rates were actually lower than the normal population levels. Several studies have suggested that rates would be even higher in PKU, particularly in female patients [26,61,62], but more recent studies showed no significant difference in body composition or overweight rates between PKU and healthy populations [45,63,64]. The etiology of overweight and obesity is very complex, with several contributing factors, but it is possible that in PKU this is related to unhealthy eating habits and lack of exercise similar to the general population [65].

The impact of phenotype was also investigated. One study found that physical growth was normal in PKU regardless of the phenotype but studied twice the number of patients with a mild-moderate phenotype compared with severe PKU [26]. Severe phenotype was more dominant than milder types in three studies that showed suboptimal growth [8,31,32]. Kennedy et al. [8] also found that length was normal during the first two years of life in milder PKU, compared to reduced length in severe PKU. The phenotype distribution was not provided in all studies; however, these findings may partly explain the discrepancy between the studies in terms of growth results. As the dietary Phe tolerance, and hence the restrictions of diet therapy, depend on the severity of the disease, phenotype distribution should always be considered in future studies assessing growth in PKU.

One of our main objectives was to evaluate the effects of gender on growth in patients with PKU, but we were unable to perform a meta-analysis examining impact of gender due to insufficient data. Although some evidence in this systematic review indicated a delayed catch-up in height and head circumference in boys [20], further studies are needed to confirm if there is an impact of gender on growth in PKU.

There are several limitations to this systematic review. Firstly, most studies with suboptimal growth outcomes presented results from patients born before 1990s [8,19,20,22,36,37]. In contrast, studies with normal growth outcomes were mostly published after 2010, and data was collected more recently (1980 to 2014) [26,27,28]. A similar cohort effect was supported by others [29]. Additionally, Thiele et al. [32] showed that growth impairment was more pronounced in children who received a casein hydrolysate during childhood, which was the major protein substitute of early years with a poor nutritional composition. Therefore, the suboptimal growth outcomes in early years of treatment can be attributed to the effects of outdated dietary practices and poor quality of early protein substitutes, rather than the effects of the Phe restricted diet or the disorder itself. More recent studies, following newer guidelines, using improved and more palatable protein substitutes provide evidence of adequate growth in PKU. Another important limitation was the high heterogeneity between studies probably arising from difference in phenotype distribution, country of study, time of data recruitment and sample sizes. Factors underlying the growth impairment in PKU were complicated due to lack of data, particularly on dietary intakes, metabolic control, and patient adherence. The duration of follow-up in majority of the studies was too short to examine the metabolic control during the whole developmental period. There was also limited information on early feeding practices (e.g., breastfeeding status, the type of protein substitutes used in infancy), parental growth, the use of adjunct treatments (such as LNAAs) or presence of any other chronic diseases which may all affect growth.

The methods of presenting growth data were variable (e.g., z-scores vs. mean values of anthropometric data, weight-for-age vs. weight-for-height, tables vs. figures). This heterogeneity led to inability to include all data in the meta-analysis. These challenges highlight the importance of using a standard data presentation. National growth standards of healthy children or healthy siblings were used for comparison of growth results with PKU, rather than using of a global reference (e.g., growth reference of World Health Organization). In future studies, interpretation of growth outcomes in PKU by using a standard growth reference as a control would allow for a valid comparison between studies conducted in different countries.

## 5. Conclusions

This is the first systematic review and meta-analysis evaluating long-term growth outcomes in PKU. Evidence suggests that even with advances in dietary treatment, “optimal” growth outcomes are not achieved in PKU. Impairment in linear growth remains an issue particularly during the early years of life and adolescence, but growth impairment is unlikely in children with mHPA. There was no evidence of a higher incidence of overweight or obesity in both patient populations during childhood or adolescence. Longitudinal data reflecting the effects of current dietary practices on growth in PKU is lacking, as most growth data is from studies on children with PKU who were born before 1990. Further studies are needed to confirm the relationship between growth and factors such as phenotype, gender, dietary intakes, and metabolic control.

## Figures and Tables

**Table 1:** Main characteristics of included studies.

Reference	Country	Study Design	Study Period	PKU Phenotype	Age Range (Years)	Gender (Male:Female)	Ethnic Origin	Duration of Follow-Up (Years)
				(Sample Size, *n*)				
Kennedy et al., 1967 [8]	USA	Prospective longitudinal	1962–1966	Classical (*n* = 27)	0–3	N/A	N/A	2 to 3
				Mild-moderate (*n* = 10)				
				Atypical (*n* = 11)				
				Total (*n* = 48)				
Hoeksma et al., 2005 [36]	The Netherlands	Retrospective longitudinal multicentre	1974–1995	N/A (Total *n* = 174)	0–3	N/A	Caucasian	3
Verkerk et al., 1994 [19]	The Netherlands	Prospective longitudinal	1974–1988	N/A (Total *n* = 137)	N/A	N/A	Caucasian	10
Schaefer et al., 1994 [20]	Germany	Prospective longitudinal	1978–1984	N/A (Total *n* = 82)	0–6	39:43	Caucasian	6
Dhondt et al., 1995 [22]	France	Retrospective longitudinal	1969–1987	N/A (Total *n* = 94)	0–10	48:46	Caucasian	10
Aldámiz-Echevarría et al., 2014 [31]	Spain	Retrospective, longitudinal, multicentre	N/A	Classical (*n* = 158)	1–36	236:269	Caucasian	PKU: 18,
				Mild-moderate (*n* = 121)				
				Mild HPA (*n* = 226)				mHPA: 12
				Total (*n* = 505)				
Thiele et al., 2017 [32]	Germany	Retrospective longitudinal	1969–2014	PKU (*n* = 183)	0–18	119:105	Caucasian	18
				Mild HPA (*n* = 41)				
				Total (*n* = 224)				
Belanger-Quintana et al., 2011 [26]	Spain	Retrospective, longitudinal, single centre	1979–2008	Classical (*n* = 34)	1–28	75:85	N/A	PKU: 18,
				Mild-moderate (*n* = 65)				
				Mild HPA (*n* = 61)				mHPA: 9
				Total (*n* = 160)				
Couce et al., 2015 [27]	Spain	Retrospective longitudinal	1980–2011	Classical (*n* = 30)	N/A	45:64	N/A	18
				Mild-moderate (*n* = 24)				
				Mild HPA (*n* = 55)				
				Total (*n* = 109)				
Evans et al., 2017 [28]	Australia	Prospective longitudinal	1996–2014	N/A (Total *n* = 32)	0.83–18	10:22	N/A	2
van der Schot et al., 1994 [37]	The Netherlands	Prospective longitudinal	N/A	Classical PKU (*n* = 33)	N/A	N/A	N/A	2
Chang et al., 1984 [38]	USA	Retrospective longitudinal	1968–1977	Classical PKU (*n* = 67)	N/A	31:36	N/A	6
Kindt et al., 1983 [39]	Norway	Prospective longitudinal	1975–1979	Classical PKU (*n* = 16)	2–6	7:9	N/A	2 to 6

PKU: Phenylketonuria; N/A: Not available; mHPA: mild hyperphenylalaninemia; n= sample size.

**Table 2:** Overview of studies included in the systematic review and meta-analysis.

Reference	Comparison	Outcomes	Key Findings
Kennedy et al., 1967 [8]	N/A	Weight, height, birth weight	Two years of longitudinal prospective data showed that weight was normal at birth and 12 months, but it was approximately 1.5 SD below normal weight-for-age (50th percentile) in severe PKU patients. Length was below normal (50th percentile) at 12 and 24 months in severe PKU. In milder groups, weight and length was normal during the first 2 years of life. There was no correlation between growth parameters and dietary Phe intake or blood Phe levels.
Hoeksma et al., 2005 [36]	National Dutch Growth Study (1955–1997) ^a^	Height, HC	Median height SD was close to zero during the first 6 months, slightly decreased at 12 months then remained constant around −0.7 SD. During the first 6 months, median HC SD was lower (close to −1 SD) but after 12 months, it gradually increased and was close to zero. After adjustment for energy intake during the first year of life, HC was positively correlated with natural and total protein intake, but not with protein substitute intake. Neither protein nor energy intake was associated with height.
Verkerk et al., 1994 [19]	Dutch reference values ^b,c^, SMOCC study ^d^, and Nellhaus ^e^	Weight, height, birth weight, HC	PKU infants were smaller at birth compared with reference lengths. A further decrease in height z-score was apparent up to 3 years. No further decrease occurred thereafter, but mean height z-score remained below −0.5 SD. Weight-by-height was close to reference at any age. Mean HC z-score was below normal in the first few weeks, but it became close to zero afterwards.
Schaefer et al., 1994 [20]	First Zurich Longitudinal Growth Study ^f^	Weight, height, birth weight, HC	Height SDs declined up to 2.5 years of age, and gradually increased towards normal thereafter in both genders. However, boys regained a normal height later than girls. Mean weight-for-length SD was close to 50th percentile in both genders during 6 years follow-up. During the first year of life, HC SD decreased in boys but remained stable in girls. After the first year of life, HC remained stable at −0.35 SD in boys and 0.0 SD in girls.
Dhondt et al., 1995 [22]	Healthy French children	Weight, height, birth weight, HC	Length and HC was normal at birth, but weight was slightly reduced in PKU patients. Children with PKU were shorter than healthy children up to 8 years, when a relaxed low-Phe diet was introduced (this height deficit was significant only in patients born after 1981). After diet relaxation, there was a catch-up of growth, and both weight-for-age and height-for-age z-scores returned to normal values (mean z-score > 0).
Aldámiz-Echevarría et al., 2014 [31]	Spanish 2008 National Growth Study ^g1,g2^	Weight, height, BMI, birth weight	Physical growth was impaired in patients with PKU. Weight and height/length decreased below ‘z score=0′ particularly during the first 2 years of life and by adulthood. BMI was close to zero in both genders during the study period, however in females there was a sharp increase in mean BMI at age 8, and it was well above 0 SD at 18 years. Growth was normal in mHPA patients. Overweight rates were below reference values in both PKU and mHPA patients. Height and weight was positively associated with Phe intake, but not with protein intakes or blood Phe levels.
Thiele et al., 2017 [32]	Healthy German children ^h1,h2^	Weight, height, BMI, birth weight	Children with PKU were significantly shorter than healthy population from birth to 18 years of age. Women and men missed their target height by 3 cm and 5 cm, respectively. Growth impairment was more pronounced in children receiving a casein hydrolysate rather than an amino acid mixture during childhood. Growth rates were significantly reduced particularly during first 2 years and in puberty. Patients with mHPA had a decreased height SD during the first 6 years, and a lower initial growth rate compared to healthy children, which was normalized after the first year of life. Weight of patients with PKU was significantly lower from healthy peers after the age of 2 years, but was normal in mHPA. BMI was normal during the follow-up in both PKU and mHPA groups.
Belanger-Quintana et al., 2011 [26]	Healthy Spanish children	Weight, height, BMI, birth weight	Physical growth (weight, height, BMI) was normal in children with PKU or mHPA, regardless of the phenotype from birth to 18 years. Many patients with severe PKU were overweight after the end of puberty. Final height of PKU patients was 2 to 4 cm higher than their expected mean family height, but was similar to the general population.
Couce et al., 2015 [27]	N/A ^i^	Weight, height, birth weight, BMI	Long term evaluation of weight, height and BMI z scores showed normal growth in PKU and mHPA. Weight and BMI were slightly higher in comparison to reference population but did not reach statistical significance. In PKU patients, height was slightly lower compared to the healthy population but the difference was less than 1 SD. Height growth was accelerated up to 8 years in female PKU patients which leads to a slightly lower final height.
Evans et al., 2017 [28]	CDC 2002, Healthy siblings	Weight, height, BMI, body fat mass, fat free mass	Two years of longitudinal prospective growth and body composition data showed normal growth in PKU cohort compared to healthy siblings. There was no significant association between anthropometrics and dietary variables, but fat mass was negatively correlated with total and natural protein intake. A safe protein: energy ratio of 3.0–4.5 g protein/100 kcal was significantly associated with optimal growth outcomes.
van der Schot et al., 1994 [37]	N/A	Weight, height, HC	Weight, length and head circumference values were all normal at 1 month, showing a decline below the normal values at 1 year of age, and returned to normal at 2 years of age. No association was found between blood Phe levels and growth parameters.
Chang et al., 1984 [38]	N/A ^j^	Weight, height, HC	Physical growth (weight, height/length and HC) was normal in children with PKU from infancy up to six years of age compared to the general healthy population.
Kindt et al., 1983 [39]	Healthy French children ^k1,k2^	Weight, height, birth weight, HC	The effects of two different protein recommendations (RDA vs. FAO) on growth was evaluated. HC was in the normal range in both groups. During the first year of life, weight and height/length was satisfactory in both groups. The children in the RDA group followed their percentiles closely after 1 year of age. Two children in the FAO group had a decline in linear growth between 2 to 3 years of age, and one child had a decline in linear growth at 2 years. After 1 year of age, weight gain was also satisfactory in both groups.

PKU: Phenylketonuria; N/A: Not available; Phe: phenylalanine; mHPA: mild Hyperphenylalaninemia; HC: head circumference; BMI: body mass index; SD: standard deviation. FAO: Food and Agriculture Organization; RDA: recommended dietary allowance; CDC: Centers for Disease Control and Prevention. References of growth data: ^a^ Fredriks AM, et al. Pediatr Res 2000; 47:316–23; ^b^ Kloosterman GJ. Tijdschr Kindergeneeskd 1969; 37:209-25; ^c^ Roede MJ, et al. TSoc Gezondheidsz 1985; 63:1-34; ^d^ Herngreen WP, et al. Eur J Public Health 1992; 2:117-22; ^e^ Nellhaus G. Pediatrics 1968; 41:106-14; ^f^ Prader A, et al. Helv Paediatr Acta 1989; (Supple 52):1-125; ^g1^ Carrascosa Lezcano A, et al. Pediatr (Barc) 2008;68:544e51; and ^g2^ Carrascosa Lezcano A, et al. Pediatr (Barc) 2008;68:552e69; ^h1^ Brandt I, et al. Klin Padiatr 1988;200(6):451–6, and ^h2^ Kromeyer-Hauschild K, et al. Mschr Kinderheilk 2001;149(8):807–18; (^i^) Carrascosa A, et al. Endocrinol Nutr 2008; 55:484–506; ^j^ Vaughan VC III: Developmental pediatrics, in Nelson WE, Vaughan VC, McKay JR (eds): Textbook of Pediatrics, 10th ed. Philedelphia, WB Saunders, 1975, pp 40-47; ^k1^ Sundal A. The norms for height and weight in healthy Norwegian children from birth to 15 years of age. Bergen, Norway: Grieg, 1957, and ^k2^ Karlberg P, et al. Acta Paediatr Scand Suppl 1976;258:7-76.

**Table 3:** Quality of studies according to Grading of Recommendations Assessment, Development and Evaluation (GRADE) system.

Outcomes	NoS	Study Design	RoB	Inconsistency	Indirectness	Imprecision	Publication Bias
Birth weight	8	Observational	Not serious	Not serious	Not serious	Not serious	Not likely
Weight	12	Observational	Not serious	Serious	Not serious	Not serious	Not likely
Length/height	13	Observational	Not serious	Serious	Not serious	Not serious	Not likely
BMI	5	Observational	Not serious	Not serious	Not serious	Not serious	Not likely
HC	7	Observational	Not serious	Serious	Not serious	Not serious	Not likely
Blood Phe	7	Observational	Not serious	Not serious	Not serious	Not serious	Not likely

NoS: number of studies; RoB: risk of bias; BMI: body mass index; HC: head circumference; Phe: phenylalanine.

**Table 4:** Risk of bias assessment according to the ROBINS-I tool.

Reference	D1	D2	D3	D4	D5	D6	D7	Overall
Kennedy et al., 1967 [8]	2	1	1	1	1	1	1	2—Moderate
Hoeksma et al., 2005 [36]	2	1	1	1	1	1	1	2—Moderate
Verkerk et al., 1994 [19]	2	1	1	1	1	1	1	2—Moderate
Schaefer et al., 1994 [20]	2	1	1	1	1	1	1	2—Moderate
Dhondt et al., 1995 [22]	2	1	1	4	1	1	1	4—Critical
Aldámiz-Echevarría et al., 2014 [31]	2	1	1	1	1	1	1	2—Moderate
Thiele et al., 2017 [32]	2	1	1	2	1	1	1	2—Moderate
Belanger-Quintana et al., 2011 [26]	2	1	1	1	1	1	1	2—Moderate
Couce et al., 2015 [27]	2	1	1	1	1	1	1	2—Moderate
Evans et al., 2017 [28]	2	1	1	1	1	1	1	2—Moderate
van der Schot et al., 1994 [37]	2	1	1	1	1	1	1	2—Moderate
Chang et al., 1984 [38]	2	1	1	1	1	1	1	2—Moderate
Kindt et al., 1983 [39]	2	1	1	1	1	1	1	2—Moderate

D: Domain; D 1: confounding; D2: selection of participants; D3: classification of intervention; D4: deviation from interventions; D5: missing outcome data; D6: measurement of outcomes; D7: selection of reported result; Overall. Risk of bias assessment: 0—No information; 1—Low; 2—Moderate; 3—Serious; 4—Critical.

**Table 5:** Meta-analyses of height-for-age z-scores in children with phenylketonuria.

Age (Year)	NoS	NoP Tested	Effect Size	Heterogeneity *I*^2^, *P*	95% CI	*P*
0	3 ^a^	488	−0.192	*I*^2^ = 93.2%, *P* = 0.0001	−0.466 to +0.081	0.168
0.5	2 ^b^	361	−0.157	*I*^2^ = 97.1%, *P* = <0.0001	−0.447 to +0.133	0.290
1	3 ^a^	459	−0.422	*I*^2^ = 96.7%, *P* = <0.0001	−0.672 to −0.173	<0.001
1.5	2 ^b^	361	−0.629	*I*^2^ = 85.8%, *P* = 0.008	−0.735 to −0.522	<0.0001
2	3 ^a^	500	−0.646	*I*^2^ = 90.9%, *P* = <0.0001	−0.787 to −0.505	<0.0001
2.5	2 ^b^	361	−0.661	*I*^2^ = 55.78%, *P* = 0.133	−0.718 to −0.603	<0.0001
3	3 ^a^	505	−0.566	*I*^2^ = 72.9%, *P* = 0.014	−0.649 to −0.484	<0.0001
3.5	2 ^b^	361	−0.470	*I*^2^ = 63.3%, *P* = 0.099	−0.543 to −0.397	<0.0001
4	3 ^a^	497	−0.493	*I*^2^ = 0.0%, *P* = 0.819	−0.519 to −0.468	<0.0001
4.5	2 ^b^	361	−0.539	*I*^2^ = 80.3%, *P* = 0.024	−0.630 to −0.447	<0.0001
5	3 ^a^	497	−0.467	*I*^2^ = 22.0%, *P* = 0.184	−0.507 to −0.427	<0.0001
5.5	2 ^b^	361	−0.439	*I*^2^ = 74.1%, *P* = 0.050	−0.519 to −0.360	<0.0001
6	3 ^a^	491	−0.462	*I*^2^ = 90.4%, *P* = 0.015	−0.605 to −0.320	<0.0001
7	2^c^	409	−0.464	*I*^2^ = 24.8%, *P* = 0.249	−0.535 to −0.393	<0.0001
8	2 ^c^	399	−0.444	*I*^2^ = 69.2%, *P* = 0.071	−0.598 to −0.291	<0.0001
9	2 ^c^	400	−0.491	*I*^2^ = 94.3%, *P* = <0.0001	−0.843 to −0.139	0.006
10	2 ^c^	399	−0.400	*I*^2^ = 91.3%, *P* = 0.0007	−0.672 to −0.127	0.004
11	2^c^	376	−0.286	*I*^2^ = 73.4%, *P* = 0.053	−0.460 to −0.112	<0.001
12	2 ^c^	388	−0.338	*I*^2^ = 63.0%, *P* = 0.100	−0.479 to −0.197	<0.0001
13	2 ^c^	372	−0.376	*I*^2^ = 0.0%, *P* = 0.381	−0.413 to −0.339	<0.0001
14	2 ^c^	379	−0.438	*I*^2^ = 50.4%, *P* = 0.156	−0.550 to −0.327	<0.0001
15	2 ^c^	365	−0.450	*I*^2^ = 66.7%, *P* = 0.083	−0.608 to −0.292	<0.0001
16	2 ^c^	374	−0.524	*I*^2^ = 0.0%, *P* = 0.541	−0.562 to −0.486	<0.0001
17	2 ^c^	362	−0.642	*I*^2^ = 0.0%, *P* = 0.946	−0.688 to −0.597	<0.0001
18	2 ^c^	352	−0.842	*I*^2^ = 0.0%, *P* = 0.863	−0.906 to −0.777	<0.0001

NoS: number of studies; NoP: number of patients; CI: confidence interval. References ^a^ Schaefer et al., 1994 [20], Aldámiz-Echevarría et al., 2014 [31], Thiele et al., 2017 [32]; ^b^ Schaefer et al., 1994 [20], Aldámiz-Echevarría et al., 2014 [31]; ^c^ Aldámiz-Echevarría et al., 2014 [31], Thiele et al., 2017 [32].

**Table 6:** Meta-analyses of weight-for-age z-scores in children with phenylketonuria.

Age (Year)	NoS ^a^	NoP Tested	Effect Size	Heterogeneity *I*^2^, *P*	95% CI	*P*
0	2	406	−0.090	*I*^2^ = 0.0%, *P* = 0.318	−0.192 to +0.012	0.085
1	2	377	−0.318	*I*^2^ = 92.5%, *P* = <0.001	−0.641 to +0.006	0.054
2	2	418	−0.391	*I*^2^ = 57.4%, *P* = 0.126	−0.506 to −0.275	<0.0001
3	2	423	−0.282	*I*^2^ = 0.0%, *P* = 0.836	−0.309 to −0.256	<0.0001
4	2	415	−0.224	*I*^2^ = 41.3%, *P* = 0.192	−0.314 to −0.135	<0.0001
5	2	415	−0.276	*I*^2^ = 91.6%*, P =* <0.001	−0.555 to +0.002	0.052

NoS: number of studies; NoP: number of patients; CI: confidence interval. References: ^a^ Aldámiz-Echevarría et al., 2014 [31], Thiele et al., 2017 [32].

**Table 7:** Meta-analyses of height-for-age z-scores in children with mild hyperphenylalaninemia.

Age (Year)	NoS ^a^	NoP Tested	Effect Size	Heterogeneity *I*^2^, *P*	95% CI	*P*
0	2	256	−0.161	*I*^2^ = 93.4%, *P <* 0.0001	−0.780 to +0.458	0.610
1	2	257	−0.236	*I*^2^ = 86.7%, *P* = 0.006	−0.588 to +0.116	0.189
2	2	255	−0.255	*I*^2^ = 75.6%, *P* = 0.043	−0.541 to 0.031	0.081
3	2	252	−0.140	*I*^2^ = 0.0%, *P* = 0.477	−0.184 to −0.096	<0.0001
4	2	253	−0.298	*I*^2^ = 88.4%, *P* = 0.003	−0.775 to +0.180	0.222
5	2	253	−0.246	*I*^2^ = 91.9%, *P* = 0.0005	−0.889 to +0.397	0.453
6	2	247	−0.183	*I*^2^ = 86.7%, *P* = 0.006	−0.730 to +0.365	0.513
7	2	243	−0.047	*I*^2^ = 89.0%, *P* = 0.003	−0.846 to +0.753	0.909
8	2	240	−0.123	*I*^2^ = 75.6%, *P* = 0.043	−0.719 to +0.473	0.687
9	2	238	−0.069	*I*^2^ = 71.2%, *P* = 0.063	−0.617 to +0.478	0.804
10	2	234	−0.333	*I*^2^ = 78.4%, *P* = 0.031	−1.124 to +0.458	0.409
11	2	235	−0.134	*I*^2^ = 47.9%, *P* = 0.166	−0.540 to +0.273	0.519
12	2	234	−0.288	*I*^2^ = 0.0%, *P* = 0.980	−0.385 to −0.191	<0.0001

NoS: number of studies; NoP: number of patients; CI: confidence interval. References: ^a^ Aldámiz-Echevarría et al., 2014 [31], Thiele et al., 2017 [32].

**Table 8:** Meta-analyses of weight-for-age z-scores in children with mild hyperphenylalaninemia.

Age (Year)	NoS ^a^	NoP Tested	Effect Size	Heterogeneity *I*^2^, *P*	95% CI	*P*
0	2	256	0.218	*I*^2^ = 0.0%, *P* = 0.783	+0.104 to +0.332	0.0002
1	2	257	−0.274	*I*^2^ = 0.0%, *P* = 0.683	−0.301 to −0.246	<0.0001
2	2	255	−0.319	*I*^2^ = 0.0%, *P* = 0.328	−0.358 to −0.280	<0.0001
3	2	252	−0.169	*I*^2^ = 0.0%, *P* = 0.470	−0.218 to −0.120	<0.0001
4	2	253	−0.171	*I*^2^ = 77.2%, *P* = 0.036	−0.453 to +0.110	0.233
5	2	253	−0.184	*I*^2^ = 48.7%, *P* = 0.163	−0.375 to +0.007	0.059
6	2	247	−0.059	*I*^2^ = 33.8%, *P* = 0.219	−0.253 to +0.134	0.548
7	2	243	0.129	*I*^2^ = 56.5%, *P* = 0.129	−0.207 to +0.465	0.453
8	2	240	−0.021	*I*^2^ = 0.0%, *P* = 0.504	−0.084 to +0.041	0.504
9	2	238	0.035	*I*^2^ = 0.0%, *P* = 0.553	−0.040 to +0.108	0.356
10	2	234	−0.045	*I*^2^ = 61.7%, *P* = 0.106	−0.690 to +0.601	0.892
11	2	235	0.177	*I*^2^ = 29.2%, *P* = 0.235	−0.145 to +0.498	0.282
12	2	234	0.146	*I*^2^ = 0.0%, *P* = 0.367	+0.054 to +0.237	**0.002**

NoS: number of studies; NoP: number of patients; CI: confidence interval. References: ^a^ Aldámiz-Echevarría et al., 2014 [31], Thiele et al., 2017 [32].

## Supplementary Materials

The following are available online at https://www.mdpi.com/2072-6643/11/9/2070/s1, Table S1: Timing of diet initiation and description of intervention, Table S2: Dietary intakes of the intervention group in the included studies, Table S3: Metabolic control of patients and data regarding parental growth.
