# Supplementary material for: Long-Term Growth in Phenylketonuria: A Systematic Review and Meta-Analysis
Source: Nutrients. 2019 Sep 3;11(9):2070. doi: 10.3390/nu11092070 (PMC6769966; doi:10.3390/nu11092070)
Supplement: Supplementary file 1 [file nutrients-11-02070-s001.pdf]

Supplementary Table S1. Timing of diet initiation and description of intervention.

Reference	Time of diet initiation	Description of intervention
Kennedy et al., 1967 [7]	Within 12 to 20 days of life	All PKU infants were treated with a Phe-restricted diet, supplemented with a hydrolyzed casein preparation (Lofenalac). Solids were introduced at 10-12 months. No supplements were given. Sufficient supplementary low protein food was given to fulfil energy and protein requirements.
Hoeksma et al., 2005 [19]	Within 21 days of life	N/A
Verkerk et al., 1994 [20]	Within 2 to 3 weeks of life	N/A
Schaefer et al., 1994 [21]	Within 4 to 48 days of life	Reduction of Phe intake was accomplished by the use of various Phe-free protein substitutes (both bovine serum, casein hydrolysates and Phe-free amino acid mixtures). Diet was supplemented with cow's milk and low protein foods (cereals, fruits, vegetables, low protein products). All were included in the total protein intake.
Dhondt et al., 1995 [23]	During the neonatal period	All PKU patients were treated with a Phe-restricted diet, supplemented with Lofenalac until 6 months of age, then Aminogram (patients born before 1983) or PKU 2. At 8 years of age, a relaxed diet progressively replaced the Phe-restricted diet *(blood Phe were kept between 15 and 20 mg/dl).*
Aldámiz-Echevarría et al., 2014 [26]	Within weeks or days of life	All PKU patients were treated with Phe-restricted diet, supplemented with Phe-free amino acid mixtures and low-protein foods. Patients were allowed to consume unlimited amounts of fruits and vegetables after 1996.
Thiele et al., 2017 [27]	During the neonatal period	Patients born before 1980 consumed a casein hydrolysate (Berlophen) during childhood (at least in the first 10 years of life) and patients born after 1989 were exclusively receiving Phe-free protein substitute. In 27 patients (born between 1971 and 1981) diet was interrupted at a median age of 7 years (range 6-15 years), and of these, 19 restarted therapy before adulthood (median age of 14 years)
Belanger-Quintana et al., 2011 [31]	N/A	All PKU patients were treated with a Phe-restricted diet, supplemented with Phe-free amino acid mixtures. Natural proteins in peeled potatoes, and all kind of fruits and vegetables were provided without restriction after 1994. Mild HPA patients followed a normal diet.
Couce et al., 2015 [32]	Within first month of life	All PKU patients were treated with a Phe-restricted diet, supplemented with Phe-free amino acid mixtures. Dietary treatment was held according to the Spanish PKU guidelines. Mild HPA patients followed a normal diet.

N/A: Not available; PKU: phenylketonuria; Phe: phenylalanine; HPA: hyperphenylalaninemia.

Supplementary Table S1 (cont). Timing of diet initiation and description of intervention.

Reference	Time of diet initiation	Description of intervention
Evans et al., 2017 [33]	N/A	All PKU patients were treated with a Phe-restricted diet, supplemented with Phe-free amino acid mixtures. Fruits and vegetables were allowed freely.
van der Schot et al., 1994 [39]	Within 9 to 21 days of life	N/A
Chang et al., 1984 [40]	Within first 2 months of life	N/A
Kindt et al., 1983 [41]	Within 15 to 18 days of life	The diet was based on Albumaid XP. Neglible amounts of protein from protein-free foods were excluded. The protein content of vegetables and fruits were included. Half of the patients were given a daily protein intake according to the recommendations of RDA (received about 60% more Albumaid XP than FAO), and half of the patients were followed a dietary protein intake based on the recommendations of FAO.

N/A: Not available; PKU: phenylketonuria; Phe: phenylalanine; HPA: hyperphenylalaninemia; FAO: Food and Agriculture Organization; RDA: recommended dietary allowance.

Supplementary Table S2. Dietary intakes of the intervention group in the included studies.

Reference	Dietary intakes			
	Phenylalanine (mg/kg/day)	*Natural protein (g/kg/day)*	*Protein equivalent from substitutes (g/kg/day)*	*Total protein (g/kg/day)*
Kennedy et al., 1967 [7]	*(900 mg/day in milder types)*	N/A	N/A	*(In 3 of 11 atypical patients were taking normal diets up to 90 g protein daily)*
Hoeksma et al., 2005 [19]	N/A	**0-6 months:** 1.22±0.53<br>**6-12 months:** 1.03±0.41<br>**1–2 years:** 0.97±0.46<br>**2–3 years:** 0.93±0.51<br>***Mean (0-3 years):*** 0.99±0.34	**0-6 months:** 1.46±0.58<br>**6-12 months:** 1.28±0.38<br>**1–2 years:** 1.26±0.34<br>**2–3 years:** 1.22±0.42<br>***Mean (0-3 years):*** 1.29±0.28	**0-6 months:** 2.70±0.50<br>**6-12 months:** 2.36±0.51<br>**1–2 years:** 2.25±0.56<br>**2–3 years:** 2.20±0.65<br>***Mean (0-3 years):*** 2.33±0.42
Verkerk et al., 1994 [20]	N/A	N/A	N/A	N/A
Schaefer et al., 1994 [21]	**0-2 years:** 28.3±5.0<br>**3-6 years**: 19.0±5.0	N/A	N/A	**0–2 years:** 2.24±0.5<br>**3–6 years:** 1.98±0.25
Dhondt et al., 1995 [23]	N/A	N/A	N/A	N/A
Aldámiz-Echevarría et al., 2014 [26]	**0-2 years:** 28.2 [20.8-36.2]<br>**2-9 years:** 15.5 [10.9-20.4]<br>**9-12 years:** 8.6 [7.0-11.3]<br>**12-18 years:** 7.6 [5.4-10.3]	**0-2 years:** 0.8 [0.5-1.0]<br>**2-9 years:** 0.5 [0.3-0.9]<br>**9-12 years:** 0.3 [0.2-0.5]<br>**12-18 years:** 0.3 [0.2-0.5]	**0-2 years:** 1.4 [1.0-1.7]<br>**2-9 years:** 1.3 [1.0-1.7]<br>**9-12 years:** 1.2 [0.9-1.4]<br>**12-18 years:** 1.0 [0.7-1.3]	**0-2 years:** 2.2 [1.8-2.8]<br>**2-9 years:** 1.8 [1.4-2.3]<br>**9-12 years:** 1.2 [1.0-1.7]<br>**12-18 years:** 1.1 [1.0-1.4]
Thiele et al., 2017 [27]	N/A	**0-1 year:** 0.7<br>**2-10 years:** 0.2-0.5<br>**>10 years:** 0.2	**0-1 year:** 1.6<br>**2-10 years:** 1.0-1.6<br>**>10 years:** 0.8-1.0	**0-1 year:** 2.3<br>**2-10 years:** 1.2-2.1<br>**>10 years:** 1.0-1.2

N/A: Not available.

Supplementary Table S2 (cont). Dietary intakes of the intervention group in the included studies.

Reference	Dietary intakes			
	Phenylalanine (mg/kg/day)	*Natural protein (g/kg/day)*	*Protein equivalent from substitutes (g/kg/day)*	*Total protein (g/kg/day)*
Belanger-Quintana et al., 2011 [31]	N/A	*Mild PKU:*<br>**0-2 years:** 15-25 g/day<br>**2-18 years:** 15-40 g/day<br><br>*Moderate PKU:*<br>**0-18 years:** 6-15 g/day<br><br>*Classic PKU:*<br>**0-18 years:** 0-6 g/day	*Mild-PKU:*<br>**0-2 years:** 2.5 - 3.0<br>**2-9 years:** 2.0 - 2.5<br>**9-12 years:** 1.5<br>**12-18 years:** 1.0<br>*Moderate & Classic PKU:*<br>**0-2 years:** 3.0<br>**2-9 years:** 2.5<br>**9-12 years:** 2.0<br>**12-18 years:** 1.5	*Mild-PKU:*<br>**0-2 years:** 4.0 - 6.0<br>**2-9 years:** 4.0 - 5.0<br>**9-12 years:** 3.0 - 4.0<br>**12-18 years:** 3.0 - 4.0<br>*Moderate & Classic PKU:*<br>**0-2 years:** 3.0 - 5.0<br>**2-9 years:** 3.0 - 4.0<br>**9-12 years:** 3.0 - 4.0<br>**12-18 years:** 2.0 - 3.0
Couce et al., 2015 [32]	N/A	N/A	N/A	*(The average protein intake was 1.3–1.5 times above the RDA)*
Evans et al., 2017 [33]	N/A	0.5±0.18 (0.18 - 0.80)	1.54±0.50 (0.80 - 2.70)	2.05±0.60 (1.00-3.5)
van der Schot et al., 1994 [39]	N/A	N/A	N/A	N/A
Chang et al., 1984 [40]	N/A	N/A	N/A	N/A
Kindt et al., 1983 [41]	N/A	N/A	N/A	*0-12 months:*<br>**RDA-group:** 2.4<br>**FAO-group:** 1.79<br>*12-24 months:*<br>**RDA-group:** 1.95<br>**FAO-group:** 1.33

N/A: Not available; PKU: phenylketonuria; FAO: Food and Agriculture Organization; RDA: recommended dietary allowance.

Supplementary Table S3. Metabolic control of patients and data regarding parental growth.

Reference	Metabolic control	Parental growth
Kennedy et al., 1967 [7]	*Mean serum Phe levels in Classic PKU (mg/dl):*<br>**6 months (n=20):** 6.3 (range: 0.9-19.9)<br>**12 months (n=20):** 6.4 (range: 0.5-20.3)<br>**24 months (n=13):** 10.1 (range: 20)	N/A
Hoeksma et al., 2005 [19]	N/A	N/A
Verkerk et al., 1994 [20]	N/A	N/A
Schaefer et al., 1994 [21]	*Dietary management was aimed to keep plasma Phe concentrations between 120 to 360 µmol/L. Mean plasma Phe levels were 315±103 µmol/L during the first 2 years of life, and 418±151 µmol/L during the third to sixth year.*	N/A
Dhondt et al., 1995 [23]	*Before 8 years, blood Phe levels were kept below 8 mg/dl. Diet was relaxed after 8 years with a target blood Phe level of 15-20 mg/dl.*	N/A
Aldámiz-Echevarría et al., 2014 [26]	*Target blood Phe levels were established according to the Spanish protocol (0-6 years: <360 µmol/L, 6-10 years: <480 µmol/L, and >10 years: <600 µmol/L). Mean blood Phe was significantly higher in the PKU group for both males and females (p>0.05).*	N/A
Thiele et al., 2017 [27]	*Mean plasma Phe levels (µmol/L):*<br>**1 year (n=103):** 301±153 **10 years (n=76):** 361±197<br>**2 years (n=101):** 290±151 **11 years (n=76):** 367±174<br>**3 years (n=99):** 307±170 **12 years (n=76):** 423±89<br>**4 years (n=98):** 320±168 **13 years (n=76):** 456±224<br>**5 years (n=92):** 341±189 **14 years (n=76):** 468±226<br>**6 years (n=89):** 332±197 **15 years (n=76):** 506±256<br>**7 years (n=86):** 335±191 **16 years (n=76):** 541±503<br>**8 years (n=76):** 331±170 **17 years (n=76):** 579±275<br>**9 years (n=76):** 333±165 **18 years (n=76):** 637±270	*Parents of PKU has shown no sign of reduced final height (Patients' fathers: 179.4 ± 7.9 cm, healthy German men: 174.8 ± 8.1; patients' mothers: 166.0 ± 6.2 cm, healthy German women: 166.4 ± 4.1)*

N/A: Not available; Phe: phenylalanine; PKU: phenylketonuria.

Supplementary Table S3 (cont). Metabolic control of patients and data regarding parental growth.

Reference	Metabolic control	Parental growth
Belanger-Quintana et al., 2011 [31]	N/A<br>*(Metabolic control and compliance were good in the great majority of cases until the last years of adolescence.)*	PKU patients *(N=32, who reached to their final height)* were grown between 2 and 4 cm more than their expected mean family height (p<0.001) *(within the CI of the mean family height of ±5 cm)*
Couce et al., 2015 [32]	*Target Phe levels (0-6 years: <360 μmol/L, 6-10 years: <480 μmol/L, and >10 years: <600 μmol/L) were maintained.*<br>*<6 years (μmol/L):*<br>**Male (PKU):** 287.9±50.8; **Female (PKU):** 301.5±26.7<br>**Male (mHPA):** 240.7±51.2; **Female (mHPA):** 250.1±43.8<br>*6-10 years (μmol/L):*<br>**Male (PKU):** 367.2 ± 67.1; **Female (PKU):** 363.5 ± 57.1<br>**Male (mHPA):** 270.7 ± 56.7; **Female (mHPA):** 275.6 ± 37.8<br>*>10 years (μmol/L):*<br>**Male (PKU):** 481.8 ± 106.9; **Female (PKU):** 444.3 ± 80.7<br>**Male (mHPA):** 284.0 ± 54.6; **Female (mHPA):** 310.2 ± 21.4	N/A
Evans et al., 2017 [33]	N/A	N/A
van der Schot et al., 1994 [39]	*Mean plasma Phe levels were 0.35 mmol/l (5.8 mg/dl) at first year, and 0.39 mmol/l (6.5 mg/dl) at second year of life.*	N/A
Chang et al., 1984 [40]	N/A	N/A
Kindt et al., 1983 [41]	*Metabolic control was equally well in both RDA and FAO groups according to plasma Phe levels.*<br>*Mean plasma Phe levels (0-12 months):*<br>RDA group: 303-752 μmol/L, FAO group: 376-673 μmol/L;<br>*Mean plasma Phe levels (12-24 months):*<br>RDA group: 272-642 μmol/L, FAO group: 436-545 μmol/L	N/A

N/A: Not available; CI: confidence interval; Phe: phenylalanine; PKU: phenylketonuria; mHPA: mild hyperphenylalaninemia; FAO: Food and Agriculture Organization; RDA: recommended dietary allowance.
